# Supplementary material for: Nasopharyngeal carriage of Streptococcus pneumoniae in Latin America and the Caribbean: A systematic review and meta-analysis
Source: PLoS One. 2024 May 20;19(5):e0297767. doi: 10.1371/journal.pone.0297767 (PMC11104613; doi:10.1371/journal.pone.0297767)

**S1 Table: PRISMA Checklist**

| **Section and Topic** | **Item #** | **Checklist item** | **Location where item is reported** |
| --- | --- | --- | --- |
| **TITLE** | | |  |
| Title | 1 | Identify the report as a systematic review. | 1 |
| **ABSTRACT** | | |  |
| Abstract | 2 | See the PRISMA 2020 for Abstracts checklist. | 2 |
| **INTRODUCTION** | | |  |
| Rationale | 3 | Describe the rationale for the review in the context of existing knowledge. | 3 |
| Objectives | 4 | Provide an explicit statement of the objective(s) or question(s) the review addresses. | 3, 4 |
| **METHODS** | | |  |
| Eligibility criteria | 5 | Specify the inclusion and exclusion criteria for the review and how studies were grouped for the syntheses. | 4 |
| Information sources | 6 | Specify all databases, registers, websites, organizations, reference lists and other sources searched or consulted to identify studies. Specify the date when each source was last searched or consulted. | 5 |
| Search strategy | 7 | Present the full search strategies for all databases, registers and websites, including any filters and limits used. | 5 |
| Selection process | 8 | Specify the methods used to decide whether a study met the inclusion criteria of the review, including how many reviewers screened each record and each report retrieved, whether they worked independently, and if applicable, details of automation tools used in the process. | 5 |
| Data collection process | 9 | Specify the methods used to collect data from reports, including how many reviewers collected data from each report, whether they worked independently, any processes for obtaining or confirming data from study investigators, and if applicable, details of automation tools used in the process. | 5, 6 |
| Data items | 10a | List and define all outcomes for which data were sought. Specify whether all results that were compatible with each outcome domain in each study were sought (e.g. for all measures, time points, analyses), and if not, the methods used to decide which results to collect. | 6 |
|  | 10b | List and define all other variables for which data were sought (e.g. participant and intervention characteristics, funding sources). Describe any assumptions made about any missing or unclear information. | 6 |
| Study risk of bias assessment | 11 | Specify the methods used to assess risk of bias in the included studies, including details of the tool(s) used, how many reviewers assessed each study and whether they worked independently, and if applicable, details of automation tools used in the process. | 5, 6 |
| Effect measures | 12 | Specify for each outcome the effect measure(s) (e.g. risk ratio, mean difference) used in the synthesis or presentation of results. | NR |
| Synthesis methods | 13a | Describe the processes used to decide which studies were eligible for each synthesis (e.g. tabulating the study intervention characteristics and comparing against the planned groups for each synthesis (item #5)). | 6 |
|  | 13b | Describe any methods required to prepare the data for presentation or synthesis, such as handling of missing summary statistics, or data conversions. | 6 |
|  | 13c | Describe any methods used to tabulate or visually display results of individual studies and syntheses. | 6 |
|  | 13d | Describe any methods used to synthesize results and provide a rationale for the choice(s). If meta-analysis was performed, describe the model(s), method(s) to identify the presence and extent of statistical heterogeneity, and software package(s) used. | 6 |
|  | 13e | Describe any methods used to explore possible causes of heterogeneity among study results (e.g. subgroup analysis, meta-regression). | 6 |
|  | 13f | Describe any sensitivity analyses conducted to assess robustness of the synthesized results. | NR |
| Reporting bias assessment | 14 | Describe any methods used to assess risk of bias due to missing results in a synthesis (arising from reporting biases). | 5, 6 |
| Certainty assessment | 15 | Describe any methods used to assess certainty (or confidence) in the body of evidence for an outcome. | 5, 6 |
| **RESULTS** | | |  |
| Study selection | 16a | Describe the results of the search and selection process, from the number of records identified in the search to the number of studies included in the review, ideally using a flow diagram. | 7, fig 1 |
|  | 16b | Cite studies that might appear to meet the inclusion criteria, but which were excluded, and explain why they were excluded. | S2 table |
| Study characteristics | 17 | Cite each included study and present its characteristics. | Table 1 |
| Risk of bias in studies | 18 | Present assessments of risk of bias for each included study. | S4- 6 table |
| Results of individual studies | 19 | For all outcomes, present, for each study: (a) summary statistics for each group (where appropriate) and (b) an effect estimate and its precision (e.g. confidence/credible interval), ideally using structured tables or plots. | 12, fig 2- 6 |
| Results of syntheses | 20a | For each synthesis, briefly summarize the characteristics and risk of bias among contributing studies. |  |
|  | 20b | Present results of all statistical syntheses conducted. If meta-analysis was done, present for each the summary estimate and its precision (e.g. confidence/credible interval) and measures of statistical heterogeneity. If comparing groups, describe the direction of the effect. | Fig 2- 6, S3 table, S1- S8 fig |
|  | 20c | Present results of all investigations of possible causes of heterogeneity among study results. | NR |
|  | 20d | Present results of all sensitivity analyses conducted to assess the robustness of the synthesized results. | NR |
| Reporting biases | 21 | Present assessments of risk of bias due to missing results (arising from reporting biases) for each synthesis assessed. | NR |
| Certainty of evidence | 22 | Present assessments of certainty (or confidence) in the body of evidence for each outcome assessed. | NR |
| **DISCUSSION** | | |  |
| Discussion | 23a | Provide a general interpretation of the results in the context of other evidence. | 13 |
|  | 23b | Discuss any limitations of the evidence included in the review. | 13, 14 |
|  | 23c | Discuss any limitations of the review processes used. | 15 |
|  | 23d | Discuss implications of the results for practice, policy, and future research. | 15 |
| **OTHER INFORMATION** | | |  |
| Registration and protocol | 24a | Provide registration information for the review, including register name and registration number, or state that the review was not registered. | 4 |
|  | 24b | Indicate where the review protocol can be accessed, or state that a protocol was not prepared. | NR |
|  | 24c | Describe and explain any amendments to information provided at registration or in the protocol. | NR |
| Support | 25 | Describe sources of financial or non-financial support for the review, and the role of the funders or sponsors in the review. | 16 |
| Competing interests | 26 | Declare any competing interests of review authors. | 16 |
| Availability of data, code and other materials | 27 | Report which of the following are publicly available and where they can be found: template data collection forms; data extracted from included studies; data used for all analyses; analytic code; any other materials used in the review. | NR |

**S1 Annex Search strategy**

1. **MEDLINE PubMed:**

Date of search: 27/12/2022

#1 Pneumococcal Infections[Mesh]

#2 Streptococcus Pneumoniae[Mesh]

#3 Pneumococc*[tiab]

#4 IPD[tiab]

#5 Pneumococcal Vaccines[Mesh]

#6 Pnu-Imune Vaccine*[tiab]

#7 PnuImune Vaccine*[tiab]

#8 Pneumovax[tiab]

#9 PCV 10[tiab]

#10 PCV 13[tiab]

#11 PCV 15[tiab]

#12 PPV23[tiab]

#13 #1 OR #2 OR #3 OR #4 OR #5 OR #6 OR #7 OR #8 OR #9 OR #10 OR #11 OR #12

(Americas[Majr] OR Latin America[Mesh] OR Latin America*[tiab] OR Latinamerica*[tiab] OR Latinoamerica*[tiab] OR Hispanoamerica*[tiab] OR Iberoamerica*[tiab] OR Ibero Americ*[tiab] OR Panamerican*[tiab] OR Central America[Mesh] OR Central America*[tiab] OR Centroamerica*[tiab] OR Mesoamerica*[tiab] OR Meso America*[tiab] OR Middle America*[tiab] OR South America[Mesh] OR South America*[tiab] OR Southamerica*[tiab] OR Sudamerica*[tiab] OR &quot;America del Sur&quot;[tiab] OR Caribbean Region[Mesh] OR Caribbean[tiab] OR Caribe*[tiab] OR West Indies[Mesh] OR West Indi*[tiab] OR Antill*[tiab] OR Indians, South American[Mesh] OR Indians, Central American[Mesh] OR Amerindian*[tiab] OR Indians[tiab] OR American Indian*[tiab] OR Native America*[tiab] OR Patagoni*[tiab] OR Andes[tiab] OR Andean*[tiab] O Amazon*[tiab] OR Anguilla[ad] OR Anguill*[tiab] OR Anguilla[pl] OR &quot;Antigua and Barbuda&quot;[ad] OR &quot;Antigua and Barbuda&quot;[tiab] OR &quot;Antigua and Barbuda&quot;[pl] OR Argentin*[ad] OR Argentin*[tiab] OR Argentina[pl] O Bahama*[ad] OR Baham*[tiab] OR Bahama*[pl] OR Bermud*[ad] OR Bermud*[tiab] OR Bermud*[pl] OR Bolivia*[ad] OR Bolivia*[tiab] OR Bolivia[pl] OR Brazil*[ad] OR Brasil*[ad] OR Brazil*[tiab] OR Brasil*[tiab] OR Brazil[pl] OR Cayman*[ad] OR Cayman*[tiab] OR Cayman*[pl] OR Curaçao[ad] OR Curaçao[tiab] OR Curaçao[pl] OR Colombia*[ad] OR Colombia*[tiab] OR Colombia[pl] OR Chile*[ad] OR Chile*[tiab] OR Chile[pl] OR Ecuador*[ad] OR Ecuator*[ad] OR Ecuador*[tiab] OR Ecuador[pl] OR Grenad*[ad] OR Grenad*[tiab] OR Grenad*[pl] OR Guadeloup*[ad] OR Guadeloup*[tiab] OR Guadeloup*[pl]OR Guiana*[ad] OR Guiana*[tiab] OR French Guiana[pl] OR Guyan*[ad] OR Guyan*[tiab] OR Guyana[pl] OR Paraguay*[ad] OR Paraguay*[tiab] OR Paraguay[pl] OR Peru*[ad] OR Peru*[tiab] OR Peru[pl] OR Surinam*[ad] OR Surinam*[tiab] OR Surinam*[pl] OR Uruguay*[ad] OR Uruguay*[tiab] OR Uruguay[pl] OR Venez*[ad] OR Venez*[tiab] OR Venezuela[pl] OR Belize*[ad] OR Belize*[tiab] OR Belize[pl] OR Costa Ric*[ad] OR Costarric*[ad] OR Costaric*[ad] OR Costa Ric*[tiab] OR Costarric*[tiab] OR Costaric*[tiab] OR Costa Rica[pl] OR Salvador*[ad] OR Salvador*[tiab] OR El Salvador[pl] OR Guatemal*[ad] OR Guatemal*[tiab] OR Guatemala[pl] OR Hondur*[ad] OR Hondur*[tiab] O Honduras[pl] OR Martinique[ad] OR Martiniqu*[tiab] OR Martinique[pl] O Nicaragu*[ad] OR Nicaragu*[tiab] OR Nicaragua[pl] OR Panam*[ad] OR Panam*[tiab] OR Panama[pl] OR Mexico[Mesh] OR Mexic*[ad] OR Mexic*[tiab] OR Mejic*[tiab] OR Mexico[pl] OR Montserrat[ad] OR Montserrat*[tiab] OR Montserrat[pl] O Baham*[ad] OR Baham*[tiab] OR Bahamas[pl] OR Cuba*[ad] OR Cuba*[tiab] OR Cuba[pl] OR Dominic*[ad] OR Dominic*[tiab] OR Dominican Republic[pl] OR Haiti*[ad] OR Haiti*[tiab] OR Haiti[pl] OR Jamaic*[ad] OR Jamaic*[tiab] OR Jamaica[pl] OR Puerto Rico[Mesh] OR Puerto Ric*[tiab] OR Puertorric*[tiab] OR Puertoric*[tiab] OR Saint Kitts[ad] OR Saint Kitts[tiab] OR Saint Kitts[pl] OR &quot;Trinidad and Tobago&quot;[tiab] OR &quot;Trinidad and Tobago&quot;[ad] OR &quot;Trinidad and Tobago&quot;[pl])

1. **EMBase (Ovid)**

Date of search: 27/12/2022

1 exp pneumococcal infection/

2 exp Streptococcus pneumoniae/

3 Pneumococc*.ti,ab.

4 IPD.ti,ab.

5 exp Pneumococcus vaccine/

6 (Pnu-Imune adj3 Vaccine*).ti,ab.

7 (PnuImune adj3 Vaccine*).ti,ab.

8 Pneumovax.ti,ab.

9 PCV-10.ti,ab.

10 PCV-13.ti,ab.

11 PCV-15.ti,ab.

12 PPV23.ti,ab.

13 or/1-12

14 exp Latin America/ or exp South America/ or exp Central America/ or (Latin adj1 America*).ti,ab. or Latinamerica*.ti,ab. or Latinoamerica*.ti,ab. or Hispanoamerica.ti,ab. or Iberoamerica*.ti,ab. or (Ibero adj1 Americ*).ti,ab. or Panamerica*.ti,ab. or (South adj1 America*).ti,ab. or Southamerica*.ti,ab. or Sudamerica*.ti,ab. or (America adj1 Sur).ti,ab. or (Central adj1 America*).ti,ab. or Centroamerica*.ti,ab. or Mesoamerica*.ti,ab. or (Meso adj1 America*).ti,ab. or (Middle adj1 America*).ti,ab. or exp Caribbean Islands/ or Caribbean*.ti,ab. or Caribe*.ti,ab. or (West adj1 Indi*).ti,ab. or Antill*.ti,ab. or exp American indian/ or Amerindian*.ti,ab. or Indians.ti,ab. or (Native adj1 America*).ti,ab. or Patagoni*.ti,ab. or Andes.ti,ab. or Andean*.ti,ab. or Amazon*.ti,ab. or exp Argentina/ or Argentin*.ti,ab. or exp Bolivia/ or Bolivia*.ti,ab. or exp Brazil/ or Brazil*.ti,ab. or Brasil*.ti,ab. or exp Colombia/ o Colombia*.ti,ab. or exp Chile/ or Chile*.ti,ab. or exp Ecuador/ or Ecuador*.ti,ab. or exp French Guiana/ or Guiana*.ti,ab. or exp Guyana/ or Guyan*.ti,ab. or exp Paraguay/ or Paraguay*.ti,ab. or exp Peru/ or Peru*.ti,ab. or exp Suriname/ or Surinam*.ti,ab. or exp Uruguay/ or Uruguay*.ti,ab. or exp Venezuela/ or Venez*.ti,ab. or exp Belize/ or Beliz*.ti,ab. or exp Costa Rica/ or (Costa adj1 Rica).ti,ab. or Costarric*.ti,ab. or Costaric*.ti,ab. or exp El salvador/ or Salvador*.ti,ab. or exp Guatemala/ or Guatemal*.ti,ab. or exp Honduras/ or Hondur*.ti,ab. or exp Nicaragua/ or Nicaragu*.ti,ab. or exp Panama/ or Panam*.ti,ab. or exp Mexico/ or Mexic*.ti,ab. or exp Cuba/ or Cuba*.ti,ab. or exp Dominican Republic/ or Dominica*.ti,ab. or exp Haiti/ or Haiti*.ti,ab. or exp Jamaica/ or Jamaic*.ti,ab. or exp Puerto Rico/ or (Puerto adj1 Ric*).ti,ab. or Puertoric*.ti,ab. or Puertorric*.ti,ab.

15 13 and 14

16 limit 15 to yr=&quot;2000 -Current&quot;

1. **EconLIT (Ovid)**

Date of search: 27/12/2022

1 Pneumococc*.

2 IPD.ti,ab.

3 (Pneumococ* adj3 Vaccin*).

4 (Pnu-Imune adj3 Vaccine*).

5 (PnuImune adj3 Vaccine*).

6 Pneumovax.mp.

7 PCV-10.ti,ab.

8 PCV-13.ti,ab.

9 PCV-15.ti,ab.

10 PPV23.ti,ab.

11 or/1-10

12 ((Latin adj1 America*) or Latinamerica* or Latinoamerica* or Latin* or Hispanic Americans or Iberoamerica* or (Ibero adj1 Americ*) or Panamerican* or (Central adj1 America*) or Centroamerica* or Mesoamerica* or (Meso adj1 America*) or (Middle adj1 America*) or (South adj1 America*) or Southamerica* or Sudamerica* or (America adj3 Sur) or Caribbean or Caribe* or (West adj1 Indi*) or Antill* or Amerindian* or Indians or (American adj1 Indian*) or (Native* adj1 America*) or Patagoni* or Andes or Andean* or Amazon* or Argentin* or Bolivia* or Brazil* or Brasil* Colombia* or Colombia* or Colombia or Chile* or Ecuador* or Guiana* or Guyan* or Guyan* or Paraguay* or Paraguay* or Peru* or Surinam* or Surinam* or Uruguay* or Venez* or Belize* or Costa Ric* or Costarric* or Costaric* or (Costa adj1 Ric*) or Costarric* or Salvador* or Salvador* or Salvador or Guatemal* or Guatemal* or Guatemal a or Hondur* or Nicaragu* Panam* or Mexic* or Cuba* or Dominic* or Dominic* or Haiti* or Jamaic* or Puerto Ric* or Puertorric* or Puertoric*).ti,ab.

13 11 and 12

1. **CINAHL (EBSCO)**

Date of search: 27/12/2022

S14 AND S17 Limiters

S17 S15 OR S16

S16

AB (Latin N1 America*) OR Latinamerica* OR Latinoamerica* OR Latin* OR Hispanic Americans OR Iberoamerica* OR (Ibero N1 Americ*) OR Panamerican* OR (Central N1 America*) OR Centroamerica* OR Mesoamerica* OR (Meso N1 America*) OR (Middle N1 America*) OR (South N1 America*) OR Southamerica* OR Sudamerica* OR (America N1 Sur) OR Caribbean OR Caribe* OR (West N1 Indi*) OR Antill* OR Amerindian* OR Indians OR (American N1 Indian*) OR (Native N1 America*) OR Patagoni* OR Andes OR Andean* OR Amazon* OR Argentin* OR Bolivia* OR Brazil* OR Brasil* Colombia* OR Colombia* OR Colombia OR Chile* OR Ecuador* OR Guiana* OR Guyan* OR Guyan* OR Paraguay* OR Paraguay* OR Peru* OR Surinam* OR Surinam* OR Uruguay* OR Venez* OR Belize* OR (Costa N1 Ric*) OR Costarric* OR Costaric* OR Costa Ric* OR Costarric* OR Salvador* OR Salvador* OR Guatemal* OR Guatemal* OR Guatemala OR Hondur* OR Nicaragu* OR Panam* OR Mexic* OR Cuba* OR Dominic* OR Dominic* OR Haiti* OR Jamaic* OR (Puerto N1 Ric*) OR Puertorric* OR Puertoric* S15 TI (Latin N1 America*) OR Latinamerica* OR Latinoamerica* OR Latin* OR Hispanic Americans OR Iberoamerica* OR (Ibero N1 Americ*) OR Panamerican* OR (Central N1 America*) OR Centroamerica* OR Mesoamerica* OR (Meso N1 America*) OR (Middle N1 America*) OR (South N1 America*) OR Southamerica* OR Sudamerica* OR (America N1 Sur) OR Caribbean OR Caribe* OR (West N1 Indi*) OR Antill* OR Amerindian* OR Indians OR (American N1 Indian*) OR (Native N1 America*) OR Patagoni* OR Andes OR Andean* OR Amazon* OR Argentin* ORBolivia* OR Brazil* OR Brasil* Colombia* OR Colombia* OR Colombia OR Chile* OR Ecuador* OR Guiana* OR Guyan* OR Guyan* OR Paraguay* OR Paraguay* OR Peru* OR Surinam* OR Surinam* OR Uruguay* OR Venez* OR Belize* OR (Costa N1 Ric*) OR Costarric* OR Costaric* OR Costa Ric* OR Costarric* OR Salvador* OR Salvador* OR Guatemal* OR Guatemal* OR Guatemala OR Hondur* OR Nicaragu* OR Panam* OR Mexic* OR Cuba* OR Dominic* OR Dominic* OR Haiti* OR Jamaic* OR (Puerto N Ric*) OR Puertorric* OR Puertoric*

S14 S1 OR S2 OR S3 OR S4 OR S5 OR S6 OR S7 OR S8 OR S9 OR S10 OR S11 OR S12 OR S13

S13 TI PPV23 OR AB PPV23

S12 TI PCV-15 OR AB PCV-15

S11 TI PCV-13 OR AB PCV-13

S10 TI PCV-10 OR AB PCV-10

S9 TI Pneumovax OR AB Pneumovax

S8 TI (PnuImune N1 Vaccin*) OR AB (PnuImune N1 Vaccin*)

S7 TI (Pnu-Imune N1 Vaccin*) OR AB (Pnu-Imune N1 Vaccin*)

S6 TI (Pneumococcal N1 Vaccin*) OR AB (Pneumococcal N1 Vaccin*)

S5 (MH &quot;Pneumococcal Vaccine&quot;)

S4 TI IPD OR AB IPD

S3 TI Pneumococc* OR AB Pneumococc*

S2 (MH &quot;Pneumonia, Bacterial+&quot;)

S1 (MH &quot;Pneumococcal Infections+&quot;)

1. **LILACS (BVS Eng)**

Date of search: 27/12/2022

Database : LILACS

Search on : (MH Pneumococcal Infections OR MH Streptococcus Pneumoniae OR Pneumococ$ OR Neumococ$ OR IPD OR ENI OR MH Pneumococcal Vaccines O Pnu-Imune OR PnuImune OR Pneumovax OR PCV-10 OR PCV-13 OR PCV-15 OR PPV23) [Words] and 2000 OR 2001 OR 2002 OR 2003 OR 2004 OR 2005 OR 2006 OR 2007 OR 2008 OR 2009 OR 2010 OR 2011 OR 2012 OR 2013 OR 2014 OR 2015 OR 2016 OR 2017 OR 2018 OR 2019 OR 2020 OR 2021 OR 2022 [Country, year publication]

1. **Web of Science**

Date of search: 27/12/2022

(TS=Pneumococcal Infections OR TS=Streptococcus Pneumoniae OR TI=Pneumococc* OR AB= Pneumococc* OR TI=IPD OR AB=IPD OR TS=Pneumococcal Vaccines OR TI=Pnu-Imune OR AB= Pnu-Imune OR TI=PnuImune OR AB=PnuImune OR TI=Pneumovax OR AB=Pneumovax OR TI=PCV-10 OR AB=PCV-10 OR TI=PCV-13 OR AB=PCV-13 OR TI=PCV 15 OR AB=PCV 15 OR TI=PPV23 OR AB=PPV23) AND (TS=Latin America OR TI=(Latin NEAR/1 America*) OR AB=(Latin NEAR/1 America*) OR ALL=Latinamerica* OR ALL=Latinoamerica* O ALL=Hispanoamerica* OR ALL=Iberoamerica* OR TI=(Ibero NEAR/1 America*) OR AB=(Ibero NEAR/ America*) OR ALL=Panamerican* OR TS=Central America OR TI=(Central NEAR/1 America*) OR AB=(Central NEAR/1 America*) OR ALL=Centroamerica* OR ALL=Mesoamerica* OR TI=(Meso NEAR/1 America*) OR AB=(Meso NEAR/ America*) OR TI=(Middle NEAR/1 America*) OR AB=(Middle NEAR/1 America*) OR TS=South America OR TI=(South NEAR/1 America*) OR AB=(South NEAR/1 America*) OR ALL=Southamerica* OR ALL=Sudamerica* OR TI=(America NEAR/1 Sur) OR AB=(America NEAR/1 Sur) OR TS=Caribbean Region OR ALL=Caribbean OR ALL=Caribe* OR TS=West Indies OR TI=(West NEAR/1 Indi*) OR AB=(West NEAR/1 Indi*) OR ALL=Antill* OR TS=Indians, South American OR TS=Indians, Central American OR ALL=Amerindian* OR TI=(America* NEAR/3 Indian*) OR AB=(America* NEAR/3 Indian*) OR TI=(Native NEAR/1 America*) OR AB=(Native NEAR/1 America*) OR ALL=Patagoni* OR ALL=Andes OR ALL=Andean* OR ALL=Amazon* OR ALL=Anguill* OR TI=(Antigua NEAR/1 Barbuda) OR AB=(Antigua NEAR/1 Barbuda) OR ALL=Argentin* OR ALL=Baham* ALL=Bermud* OR ALL=Bolivia* OR ALL=Brazil* OR ALL=Brasil* OR ALL=Cayman* OR ALL=Curaçao OR ALL=Colombia* OR ALL=Chile* OR ALL Ecuador* OR ALL=Grenad* OR ALL=Guadeloup* OR ALL=Guiana* OR ALL=Guyan* OR ALL=Paraguay* OR ALL=Peru* OR ALL=Surinam* OR ALL=Uruguay* OR ALL=Venez* OR ALL=Belize* OR TI=(Costa NEAR/1 Ric*) OR AB=(Costa NEAR/1 Ric*) OR ALL=Costarric* OR ALL=Costaric* OR ALL=Salvador* OR ALL=Guatemal* OR ALL=Hondur* OR ALL=Martiniqu* OR ALL=Nicaragu* OR ALL=Panam* OR TS=Mexico OR ALL=Mexic* OR ALL=Montserrat* OR ALL=Cuba* OR ALL=Dominic* OR ALL=Haiti* OR ALL=Jamaic* OR TS=Puerto Rico OR TI=(Puerto NEAR/1 Ric*) OR AB=(Puerto NEAR/1 Ric*) OR ALL=Puertorric* OR TI=(Saint NEAR/1 Kitts) OR AB=(Saint NEAR/1 Kitts) O TI=(Trinidad NEAR/1 Tobago) OR AB=(Trinidad NEAR/1 Tobago)) and 2000 or 2001 or 2002 or 2003 or 2004 or 2005 or 2023 or 2022 or 2021 or 2020 o r 2019 or 2018 or 2017 or 2016 or 2015 or 2014 or 2013 or 2012 (Publication Years)

1. **Global Health (Ovid)**

Date of search: 27/12/2022

1 exp Streptococcus pneumoniae

2 Pneumococc*.ti,ab.

3 IPD.ti,ab.

4 (Pnu-Imune adj3 Vaccine*).ti,ab.

5 (PnuImune adj3 Vaccine*).ti,ab.

6 Pneumovax.ti,ab.

7 PCV-10.ti,ab.

8 PCV-13.ti,ab.

9 PCV-15.ti,ab.

10 PPV23.ti,ab.

11 or/1-10

12 exp Latin America/ or exp South America/ or exp Central America/ or (Latin adj1 America*).ti,ab. or Latinamerica*.ti,ab. or Latinoamerica*.ti,ab. or Hispanoamerica.ti,ab. or Iberoamerica*.ti,ab. or (Ibero adj1 Americ*).ti,ab. or Panamerica*.ti,ab. or (South adj1 America*).ti,ab. or Southamerica*.ti,ab. or Sudamerica*.ti,ab. or (America adj1 Sur).ti,ab. or (Central adj1 America*).ti,ab. or Centroamerica*.ti,ab. o Mesoamerica*.ti,ab. or (Meso adj America*).ti,ab. or (Middle adj1 America*).ti,ab. or exp Caribbean Islands/ or Caribbean*.ti,ab. or Caribe*.ti,ab. or (West adj1 Indi*).ti,ab. or Antill*.ti,ab. or exp American indian/ or Amerindian*.ti,ab. or Indians.ti,ab. or (Native adj1 America*).ti,ab. or Patagoni*.ti,ab. or Andes.ti,ab. or Andean*.ti,ab. o Amazon*.ti,ab. or exp Argentina/ or Argentin*.ti,ab. or exp Bolivia/ or Bolivia*.ti,ab. or exp Brazil/ or Brazil*.ti,ab. or Brasil*.ti,ab. or exp Colombia/ or Colombia*.ti,ab. or exp Chile/ or Chile*.ti,ab. or exp Ecuador/ or Ecuador*.ti,ab. or exp French Guiana/ or Guiana*.ti,ab. or exp Guyana/ or Guyan*.ti,ab. or exp Paraguay/ or Paraguay*.ti,ab. or exp Peru/ or Peru*.ti,ab. or exp Suriname/ or Surinam*.ti,ab. or exp Uruguay/ or Uruguay*.ti,ab. or exp Venezuela/ or Venez*.ti,ab. or exp Belize/ or Beliz*.ti,ab. or ex Costa Rica/ or (Costa adj1 Rica).ti,ab. or Costarric*.ti,ab. or Costaric*.ti,ab. or exp El salvador/ or Salvador*.ti,ab. or exp Guatemala/ or Guatemal*.ti,ab. or exp Honduras/ or Hondur*.ti,ab. or exp Nicaragua/ or Nicaragu*.ti,ab. or exp Panama/ or Panam*.ti,ab. or exp Mexico/ or Mexic*.ti,ab. or exp Cuba/ or Cuba*.ti,ab. or exp Dominican Republic/ or Dominica*.ti,ab. or exp Haiti/ or Haiti*.ti,ab. or exp Jamaica/ or Jamaic*.ti,ab. or exp Puerto Rico/ or (Puerto adj1 Ric*).ti,ab. or Puertoric*.ti,ab. or Puertorric*.ti,ab.

**S2 Table. List of excluded studies during full text screening process**

| **Author and year of publication** | **Reason for exclusion** |
| --- | --- |
| Andrea 2010 | Duplicate |
| Cardoso 2006 | Not enough information |
| Chavez Amaro 2018 | Duplicate |
| Chavez Amaro 2019 | Duplicate |
| Gamez 2021 | Wrong outcomes |
| Hanke 2016 | Duplicate |
| Ochoa 2001 | Duplicate |
| Pimenta 2011 | Duplicate |
| Poubel Vieira de Rezende 2021 | Wrong patient population |
| Quintero Moreno 2009 | Duplicate |
| Rivera Olivero 2009 | Duplicate |
| Rodrigues 2017 | Duplicate |
| Toledo Romani 2017 | Duplicate |
| Torres 2013 | Duplicate |

**S3 Table. Meta- analysis of nasopharyngeal carriage**

| **Nasopharyngeal carriage** | | | | | | | | | |
| --- | --- | --- | --- | --- | --- | --- | --- | --- | --- |
|  |  | **All *Spn* serotypes** | |  | **Vaccine serotypes** | | | | |
|  |  |  |  |  | **PCV10** | |  | **PCV13** | |
|  | **N studies** | **Proportion**  **(CI 95%)** | **I^2^** | **N studies** | **Proportion**  **(CI 95%)** | **I^2^** | **N studies** | **Proportion**  **(CI 95%)** | **I^2^** |
| **Overall** | 53 | 38.35% (32.96- 44.05%) | 98.40% | 49 | 33.73% (27.69- 40.36%) | 95.20% | 51 | 45.19% (37.51- 53.12%) | 97.30% |
| **by 5-years period** | | | | | | | | | |
| **1995-1999** | 8 | 40.29% (29.39- 52.24%) | 97.50% | 8 | 31.50% (13.60- 57.32%) | 97.20% | 8 | 42.04% (15.21- 74.57%) | 97.90% |
| **2000-2004** | 14 | 32.32% (24.83- 40.83%) | 97.10% | 14 | 42.45% (29.57- 56.44%) | 91.10% | 14 | 55.07% (37.50- 71.47%) | 94.60% |
| **2005-2009** | 15 | 42.37% (31.54- 53.98%) | 98.20% | 14 | 41.88% (34.68- 49.45%) | 93.20% | 15 | 48.66% (36.23- 61.25%) | 96% |
| **2010-2014** | 14 | 41.95% (28.58- 56.61%) | 98.70% | 11 | 26.15% (21.78- 31.05%) | 79.20% | 13 | 31.18% (21.19- 43.29%) | 94.60% |
| **2015-2019** | 10 | 40.48% (27.14- 55.39%) | 98% | 9 | 19.84% (8.38- 40.11%) | 95.70% | 10 | 34.00% (18.28- 54.26%) | 98.30% |
| **by age** | | | | | | | | | |
| **0-5 years** | 41 | 41.16% (34.73- 47.89%) | 98.50% | 40 | 33.99% (27.26- 41.44%) | 95.70% | 42 | 45.01% (36.05- 54.30%) | 97.70% |
| **6-64 years** | 1 | 8.19% (6.66- 10.05%) | NA | 2 | 29.82% (22.15- 38.83%) | 0% | 2 | 39.49% (30.95- 48.73%) | 0% |
| **≥ 65 years** | 1 | 26.27% (19.13- 34.93%) | NA | 1 | 51.52% (34.93- 67.77%) | NA | 1 | 57.58% (40.49- 73.02%) | NA |
| **by country** | | | | | | | | | |
| **Argentina** | 1 | 61.56% (56.42- 66.45%) | NA | 1 | 5.43% (3.11- 9.32%) | NA | 1 | 9.05% (5.91- 13.61%) | NA |
| **Barbados** | 2 | 12.09% (9.28- 15.60%) | 0% | 2 | 36.19% (22.19- 53.00%) | 27.10% | 2 | 43.89% (22.79- 67.46%) | 65.00% |
| **Bolivia** | 1 | 33.61% (29.94- 37.49%) | NA | 1 | 25.93% (16.00- 39.15%) | NA | 1 | 42.59% (30.20- 55.99%) | NA |
| **Brazil** | 22 | 36.67%(29.01%-45.06%) | 98.40% | 19 | 29.08% (18.86%-41.97%) | 96.30% | 21 | 35.44% (23.61- 49.37%) | 98.00% |
| **Chile** | 1 | 26.27%(19.13%-34.93%) | NA | 1 | 51.52% (34.93- 67.77%) | NA | 1 | 57.58% (40.49- 73.02%) | NA |
| **Colombia** | 1 | 50.56% (45.92- 55.20%) | NA | 1 | 32.59% (26.77- 39.00%) | NA | 1 | 49.11% (42.61- 55.63%) | NA |
| **Cuba** | 1 | 26.67% (24.82- 28.59%) | NA | 1 | 38.65% (34.72- 42.74%) | NA | 1 | 45.45% (34.73- 56.62%) | NA |
| **Dominican-Republic** | 1 | 61.60% (52.80- 69.70%) | NA | 1 | 28.57% (19.61- 39.61%) | NA | 1 | 65.07% (61.04- 68.90%) | NA |
| **Ecuador** | 1 | 39.88% (32.65- 47.58%) | NA | 1 | 4.35% (1.41- 12.63%) | NA | 1 | 17.39% (10.15- 28.18%) | NA |
| **Guatemala** | 2 | 36.16% (27.47- 45.86%) | 88.30% | 1 | 56.44% (48.74-6 3.85%) | NA | 1 | 69.94% (62.47- 76.48%) | NA |
| **Haiti** | 1 | 44.96% (41.27- 48.71%) | NA | 1 | 34.95% (29.84- 40.44%) | NA | 1 | 50.49% (44.93- 56.03%) | NA |
| **Jamaica** | 1 | 33.70% (28.36- 39.48%) | NA | 1 | 56.63% (45.82- 66.83%) | NA | 1 | 71.08% (60.47- 79.80%) | NA |
| **Mexico** | 6 | 33.78% (21.52- 48.68%) | 93.80% | 6 | 47.53% (34.19- 61.23%) | 93.00% | 6 | 69.00% (48.36- 84.10%) | 95.90% |
| **Peru** | 4 | 66.29% (31.58- 89.34%) | 99.70% | 4 | 37.97% (26.97- 50.36%) | 94.80% | 4 | 47.53% (36.43- 58.87%) | 94.10% |
| **Uruguay** | 1 | 54.87% (51.47- 58.23%) | NA | 1 | 21.08% (16.22- 26.92%) | NA | 1 | 31.39% (25.64- 37.77%) | NA |
| **Venezuela** | 7 | 36.29% (26.93- 46.82%) | 96.70% | 7 | 39.97% (27.12- 54.36%) | 92.40% | 7 | 53.76% (39.50- 67.43%) | 93.00% |

**S4 Table. Risk of bias assessment for cohort and cross sectional studies**

| **Author and year of**  **publication** | **Evaluation *** | | | | | | | | | | | | | | |
| --- | --- | --- | --- | --- | --- | --- | --- | --- | --- | --- | --- | --- | --- | --- | --- |
|  | **1** | **2** | **3** | **4** | **5** | **6** | **7** | **8** | **9** | **10** | **11** | **12** | **13** | **14** | **Final** |
| **Allen 2003** [[52]](https://paperpile.com/c/BGgZPq/WYJOp) | Yes | Yes | Yes | Yes | No | NA | NA | No | Yes | No | Yes | No | NA | NA | **Good** |
| **Altuzarra Hernaez 2007 [**[70]](https://paperpile.com/c/BGgZPq/03gaj) | Yes | No | CD | Yes | No | NA | NA | No | Yes | No | Yes | No | NA | NA | **Fair** |
| **Andrade 2014** [[30]](https://paperpile.com/c/BGgZPq/Lw8Hj) | Yes | Yes | CD | Yes | Yes | NA | NA | No | Yes | NA | Yes | No | NA | No | **Poor** |
| **Bello Gonzalez 2010** [[77]](https://paperpile.com/c/BGgZPq/mqad5) | Yes | Yes | CD | Yes | No | NA | NA | Yes | Yes | No | Yes | No | NA | NA | **Fair** |
| **Brandileone 2016** [[32]](https://paperpile.com/c/BGgZPq/H0bAy) | Yes | Yes | CD | Yes | Yes | NA | NA | No | Yes | No | Yes | No | NA | Yes | **Fair** |
| **Brandileone 2019** [[33]](https://paperpile.com/c/BGgZPq/G7Ucj) | Yes | Yes | CD | Yes | Yes | NA | NA | Yes | Yes | No | Yes | No | NA | No | **Poor** |
| **Cardozo 2006** [[34]](https://paperpile.com/c/BGgZPq/Igloe) | Yes | Yes | CD | Yes | No | NA | NA | No | Yes | No | Yes | No | NA | NA | **Fair** |
| **Cassiolato 2019 [**[72]](https://paperpile.com/c/BGgZPq/fDHH) | Yes | Yes | Yes | Yes | No | NA | NA | No | Yes | No | Yes | No | NA | NA | **Good** |
| **Chavez Amaro 2019 [**[47]](https://paperpile.com/c/BGgZPq/l2tr0) | Yes | Yes | No | Yes | Yes | No | Yes | No | Yes | No | Yes | No | NA | NA | **Fair** |
| **Dunn 2021** [[49]](https://paperpile.com/c/BGgZPq/etmYQ) | Yes | Yes | CD | Yes | No | Yes | No | Yes | Yes | Yes | Yes | No | Yes | yes | **Good** |
| **Espinosa de los Monteros 2007** [[53]](https://paperpile.com/c/BGgZPq/wNyfm) | Yes | Yes | CD | Yes | Yes | No | NA | No | Yes | NA | Yes | No | NA | NA | **Fair** |
| **Espinosa de los Monteros 2010** [[54]](https://paperpile.com/c/BGgZPq/6YPew) | Yes | Yes | Yes | Yes | No | Yes | No | Yes | Yes | Yes | Yes | No | Yes | NA | **Good** |
| **Espinosa de los Monteros 2019** [[71]](https://paperpile.com/c/BGgZPq/Dw6WF) | Yes | Yes | CD | Yes | No | NA | NA | No | No | NA | Yes | No | NA | No | **Poor** |
| **Fernandez 2018** [[63]](https://paperpile.com/c/BGgZPq/LIZCc) | Yes | Yes | CD | Yes | No | Yes | NA | NA | Yes | No | Yes | No | NA | No | **Poor** |
| **Franco 2010** [[35]](https://paperpile.com/c/BGgZPq/izmPX) | Yes | Yes | CD | Yes | Yes | NA | NA | No | Yes | NA | Yes | No | NA | NA | **Fair** |
| **Gentile 2017** [[27]](https://paperpile.com/c/BGgZPq/AtSFe) | Yes | Yes | CD | Yes | No | NA | NA | No | Yes | No | Yes | No | NA | No | **Poor** |
| **Gomez Barreto 2002** [[79]](https://paperpile.com/c/BGgZPq/4G7xY) | Yes | Yes | Yes | Yes | No | NA | NA | Yes | Yes | No | Yes | No | NA | No | **Fair** |
| **Grijalva 2014** [[59]](https://paperpile.com/c/BGgZPq/sZOrx) | Yes | Yes | CD | Yes | No | NA | NA | No | Yes | No | Yes | No | NA | NA | **Fair** |
| **Inverarity 2011** [[29]](https://paperpile.com/c/BGgZPq/NhQ1t) | No | Yes | CD | Yes | No | NA | NA | Yes | Yes | No | Yes | No | NA | NA | **Poor** |
| **Kumar 2020** [[28]](https://paperpile.com/c/BGgZPq/X5jlT) | Yes | No | CD | Yes | No | Yes | CD | Yes | Yes | No | Yes | No | CD | No | **Poor** |
| **Kumar 2021** [[11]](https://paperpile.com/c/BGgZPq/926Xw) | Yes | No | CD | Yes | No | Yes | NA | Yes | Yes | NA | Yes | No | NA | No | **Poor** |
| **Lamaro Cardoso 2012** [[73]](https://paperpile.com/c/BGgZPq/BQebK) | Yes | Yes | CD | Yes | No | No | NA | Yes | Yes | NA | Yes | No | NA | No | **Fair** |
| **Laranjeira 2014** [[36]](https://paperpile.com/c/BGgZPq/YKmC1) | Yes | Yes | CD | Yes | No | NA | NA | No | Yes | No | Yes | No | NA | NA | **Fair** |
| **Laval 2006** [[37]](https://paperpile.com/c/BGgZPq/bljNG) | Yes | Yes | CD | Yes | Yes | NA | NA | No | Yes | No | Yes | NA | NA | NA | **Fair** |
| **Lopes 2012** [[38]](https://paperpile.com/c/BGgZPq/SqUuf) | Yes | Yes | CD | Yes | Yes | No | NA | No | Yes | NA | Yes | No | NA | NA | **Fair** |
| **Lucarevschi 2003** [[39]](https://paperpile.com/c/BGgZPq/CqYox) | Yes | Yes | Yes | Yes | No | No | NA | No | Yes | NA | Yes | No | Yes | NA | **Fair** |
| **Mendez 2013** [[57]](https://paperpile.com/c/BGgZPq/D4xAG) | Yes | Yes | CD | Yes | No | NA | NA | No | Yes | NA | Yes | No | NA | Yes | **Fair** |
| **Menezes 2015** [[40]](https://paperpile.com/c/BGgZPq/fHGyk) | Yes | Yes | No | Yes | Yes | NA | NA | Yes | Yes | Yes | Yes | No | NA | No | **Fair** |
| **Mercado 2012** [[60]](https://paperpile.com/c/BGgZPq/dXeR1) | Yes | Yes | CD | No | Yes | No | NA | No | Yes | No | Yes | No | NA | NA | **Fair** |
| **Nelson 2018** [[61]](https://paperpile.com/c/BGgZPq/bSD5F) | Yes | Yes | CD | Yes | No | Yes | Yes | No | Yes | Yes | Yes | No | No | No | **Fair** |
| **Neves 2013** [[41]](https://paperpile.com/c/BGgZPq/iPexy) | Yes | Yes | No | Yes | No | Yes | No | Yes | Yes | No | Yes | No | No | No | **Poor** |
| **Neves 2017** [[42]](https://paperpile.com/c/BGgZPq/LtYAH) | Yes | Yes | CD | Yes | No | No | NA | Yes | Yes | NA | Yes | No | NA | No | **Fair** |
| **Neves Reis 2008** [[74]](https://paperpile.com/c/BGgZPq/5slxY) | Yes | Yes | No | Yes | No | NA | NA | No | Yes | No | Yes | No | NA | Yes | **Fair** |
| **Nicoletti 2007** [[69]](https://paperpile.com/c/BGgZPq/Sw1I8) | Yes | Yes | No | Yes | No | Yes | No | Yes | Yes | No | Yes | No | No | No | **Poor** |
| **Ochoa 2005** [[62]](https://paperpile.com/c/BGgZPq/nwpsB) | Yes | Yes | CD | Yes | No | No | NA | No | Yes | No | Yes | No | NA | NA | **Fair** |
| **Parra 2013** [[46]](https://paperpile.com/c/BGgZPq/GcYYf) | Yes | Yes | CD | Yes | No | Yes | NA | No | Yes | NA | Yes | No | NA | No | **Fair** |
| **Pinto 2019** [[75]](https://paperpile.com/c/BGgZPq/Rptjd) | Yes | Yes | CD | Yes | No | No | NA | No | Yes | NA | Yes | No | NA | No | **Fair** |
| **Quintero 2006 [**53**]** | Yes | Yes | CD | Yes | No | No | NA | No | Yes | NA | Yes | NA | NA | NA | **Fair** |
| **Quintero 2011** [[65]](https://paperpile.com/c/BGgZPq/1AWAv) | Yes | Yes | CD | Yes | No | NA | NA | Yes | Yes | No | Yes | No | NA | No | **Poor** |
| **Regalado 2021** [[76]](https://paperpile.com/c/BGgZPq/n33KY) | Yes | Yes | CD | Yes | No | NA | NA | Yes | Yes | No | Yes | No | NA | No | **Poor** |
| **Rey 2002** [[43]](https://paperpile.com/c/BGgZPq/smjfR) | Yes | Yes | No | Yes | Yes | NA | NA | No | Yes | No | Yes | No | NA | No | **Poor** |
| **Reyna 2008** [[55]](https://paperpile.com/c/BGgZPq/IdZRE) | No | Yes | CD | Yes | No | NA | NA | Yes | Yes | No | Yes | No | NA | NA | **Poor** |
| **Rivera Olivero 2007** [[66]](https://paperpile.com/c/BGgZPq/yoYYl) | Yes | Yes | Yes | Yes | No | No | NA | No | Yes | NA | Yes | No | NA | NA | **Good** |
| **Rivera Olivero 2011** [[67]](https://paperpile.com/c/BGgZPq/PBsDf) | Yes | Yes | CD | Yes | No | No | NA | No | Yes | No | Yes | No | NA | NA | **Fair** |
| **Rivera Olivero 2014** [[68]](https://paperpile.com/c/BGgZPq/zJGQo) | Yes | Yes | CD | Yes | No | NA | NA | Yes | Yes | Yes | Yes | No | NA | NA | **Fair** |
| **Silva 2022** [[44]](https://paperpile.com/c/BGgZPq/dZNQ1) | Yes | Yes | CD | Yes | No | NA | NA | No | Yes | No | Yes | No | NA | NA | **Fair** |
| **Sisco 2010** [[78]](https://paperpile.com/c/BGgZPq/qxmRH) | Yes | Yes | CD | Yes | No | NA | NA | Yes | Yes | No | Yes | No | NA | NA | **Fair** |
| **Solorzano Santos 2005** [[56]](https://paperpile.com/c/BGgZPq/8OyLW) | Yes | Yes | CD | Yes | Yes | NA | NA | Yes | Yes | No | Yes | No | NA | NA | **Fair** |
| **Toledo Romani 2017** [[48]](https://paperpile.com/c/BGgZPq/Wanec) | Yes | Yes | CD | Yes | No | NA | NA | Yes | Yes | No | Yes | No | NA | NA | **Fair** |
| **Watkins 2021** [[51]](https://paperpile.com/c/BGgZPq/HCpiS) | Yes | Yes | CD | Yes | No | NA | NA | Yes | Yes | No | Yes | No | NA | Yes | **Fair** |

* **NA**: Not applicable, **CD**: Cannot be determined

1. Was the study question or research objective clearly specified?
2. Was the study population clearly specified and defined?
3. Did at least 50% of eligible subjects take part?
4. Were all subjects screened or recruited from the same population or from similar populations (including the same period of time)? Were inclusion and exclusion criteria to take part in the study pre-specified and applied consistently to all participants?
5. Was rationale for sample size, power description or variance and effect estimations provided?
6. For analysis of this study, were the exposures of interest measured before results?
7. Was the follow-up period enough for one to reasonably expect to observe an association between exposure and result, if any?
8. For exposures that may vary in terms of amount or level, did the study examine different levels of exposure relative to the result (e.g. categories of exposure or exposure measured as a continuous variable)?
9. Were measures of exposure (independent variables) clearly defined, valid, reliable and consistently implemented for all study participants?
10. Were exposures evaluated more than once over time?
11. Were measures of results (dependent variables) clearly defined, valid, reliable and consistently implemented for all study participants?
12. Were results raters blinded to participants’ exposure?
13. Were lost to follow-up 20% or less after the study startup?
14. Were potential confounding variables key due to their impact on the exposure(s)-result(s) ratio measured and statistically adjusted?

**S5 Table. Risk of bias assessment for case series studies**

| **Author and year of publication** | **Evaluation *** | | | | | | | | | |
| --- | --- | --- | --- | --- | --- | --- | --- | --- | --- | --- |
|  | **1** | **2** | **3** | **4** | **5** | **6** | **7** | **8** | **9** | **Final** |
| **Berezin 2007 [20]** | Yes | No | CD | Yes | Yes | Yes | Yes | No | Yes | **Fair** |
| **Cullotta 2002** [[58]](https://paperpile.com/c/BGgZPq/TG7p1) | Yes | No | CD | Yes | Yes | Yes | Yes | No | Yes | **Fair** |
| **Melgar 2016** [[50]](https://paperpile.com/c/BGgZPq/6WYB4) | Yes | No | CD | Yes | Yes | Yes | Yes | No | No | **Fair** |

* **NA**: Not applicable, **CD**: Cannot be determined

1. Was the study question or objective clearly specified?
2. Was the study population clearly and fully described, including case definition?
3. Were cases consecutive?
4. Were subjects comparable?
5. Was exposure clearly described?
6. Were measures of results clearly defined, valid, reliable and consistently implemented for all study participants?
7. Was the length of follow-up appropriate?
8. Were statistical methods properly described?
9. Were results properly described?

**S6 Table. Risk of bias assessment for case-control studies**

| **Author and year of**  **publication** | **Evaluation *** | | | | | | | | | | | | |
| --- | --- | --- | --- | --- | --- | --- | --- | --- | --- | --- | --- | --- | --- |
|  | **1** | **2** | **3** | **4** | **5** | **6** | **7** | **8** | **9** | **10** | **11** | **12** | **Final** |
| **Wolf 2020 [**34] | Yes | Yes | No | Yes | Yes | Yes | No | Yes | Yes | Yes | No | No | **Fair** |

* **NA**: Not applicable, **CD**: Cannot be determined

1. Was the research question or objective in this paper clearly stated and appropriate?
2. Was the study population clearly specified and defined?
3. Did the authors include a sample size justification?
4. Were controls selected or recruited from the same or similar population that gave rise to the cases (including the same timeframe)?
5. Were the definitions, inclusion and exclusion criteria, algorithms or processes used to identify or select cases and controls valid, reliable, and implemented consistently across all study participants?
6. Were the cases clearly defined and differentiated from controls?
7. If less than 100 percent of eligible cases and/or controls were selected for the study, were the cases and/or controls randomly selected from those eligible?
8. Was there use of concurrent controls?
9. Were the investigators able to confirm that the exposure/risk occurred prior to the development of the condition or event that defined a participant as a case?
10. Were the measures of exposure/risk clearly defined, valid, reliable, and implemented consistently (including the same time period) across all study participants?
11. Were the assessors of exposure/risk blinded to the case or control status of participants?
12. Were key potential confounding variables measured and adjusted statistically in the analyses? If matching was used, did the investigators account for matching during study analysis?

**S1 Figure. Meta-analysis of PCV10 nasopharyngeal carriage**


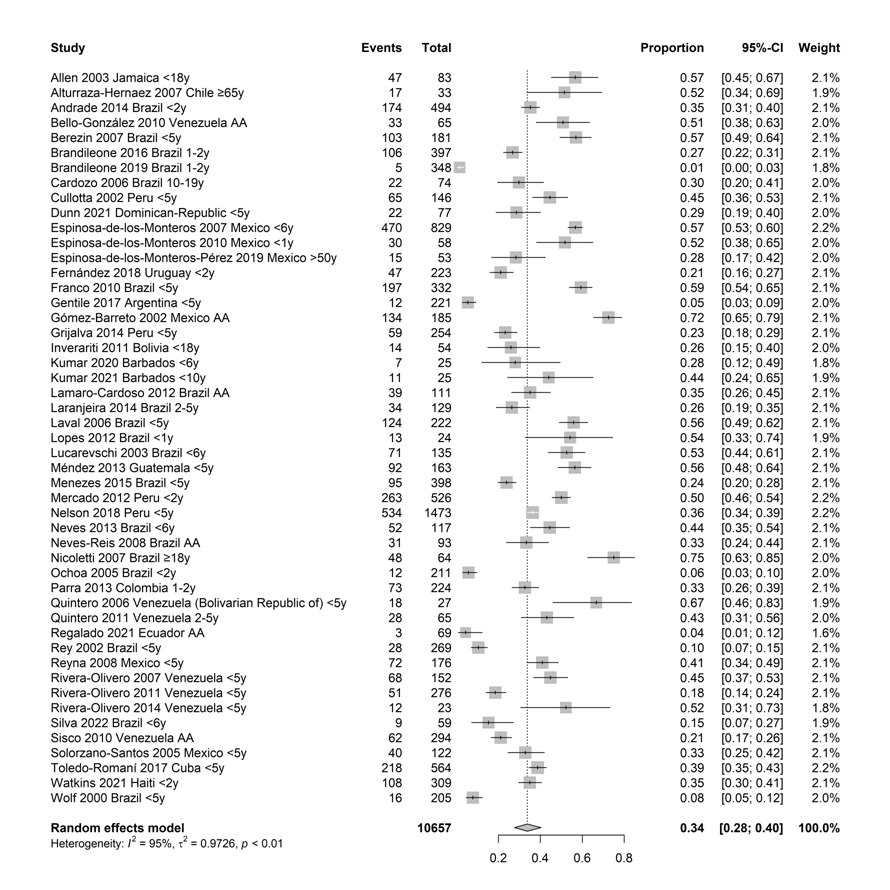


**S2 Figure. Meta-analysis of proportion of serotypes included in PCV10 nasopharyngeal carriage by five-year period**


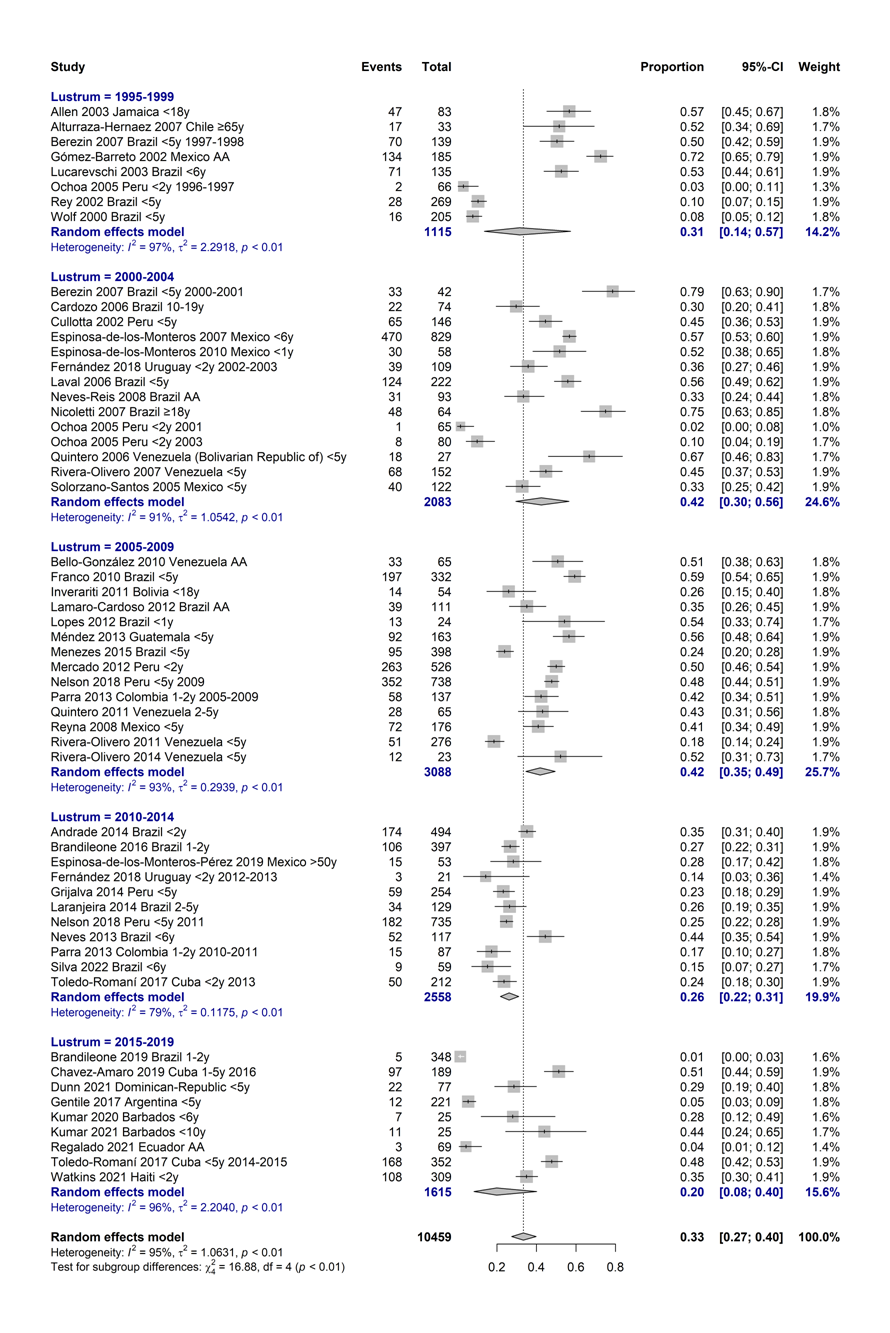


**S3 Figure. Meta-analysis of PCV10 nasopharyngeal carriage by age**


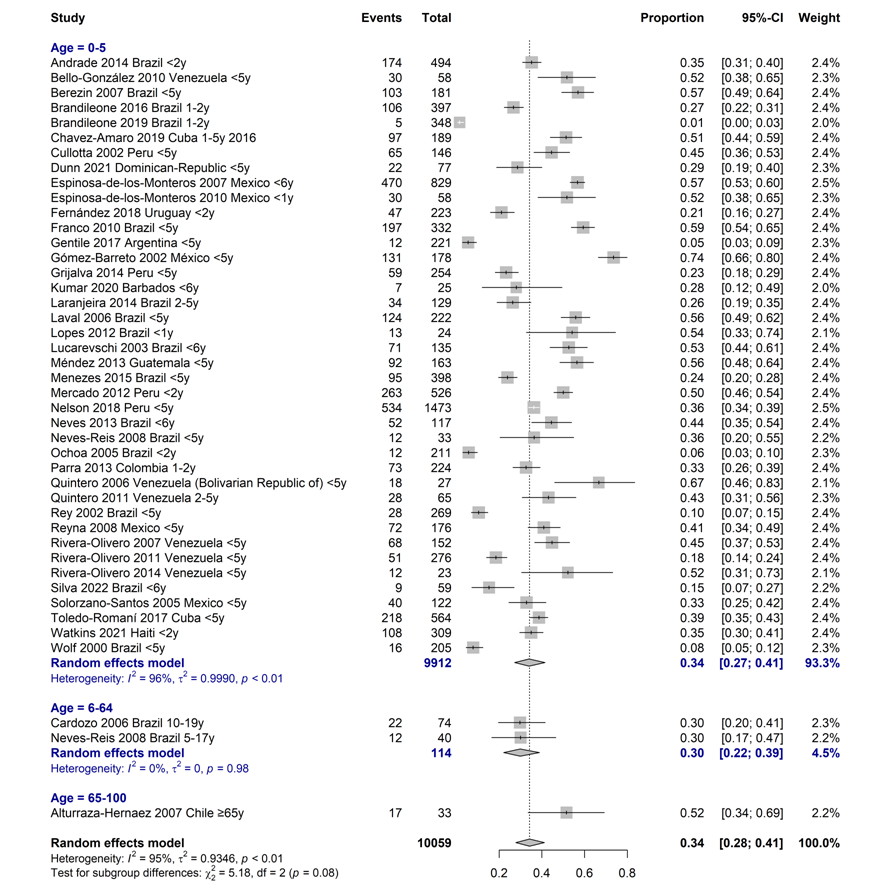


**S4 Figure. Meta-analysis of PCV10 nasopharyngeal carriage by country**


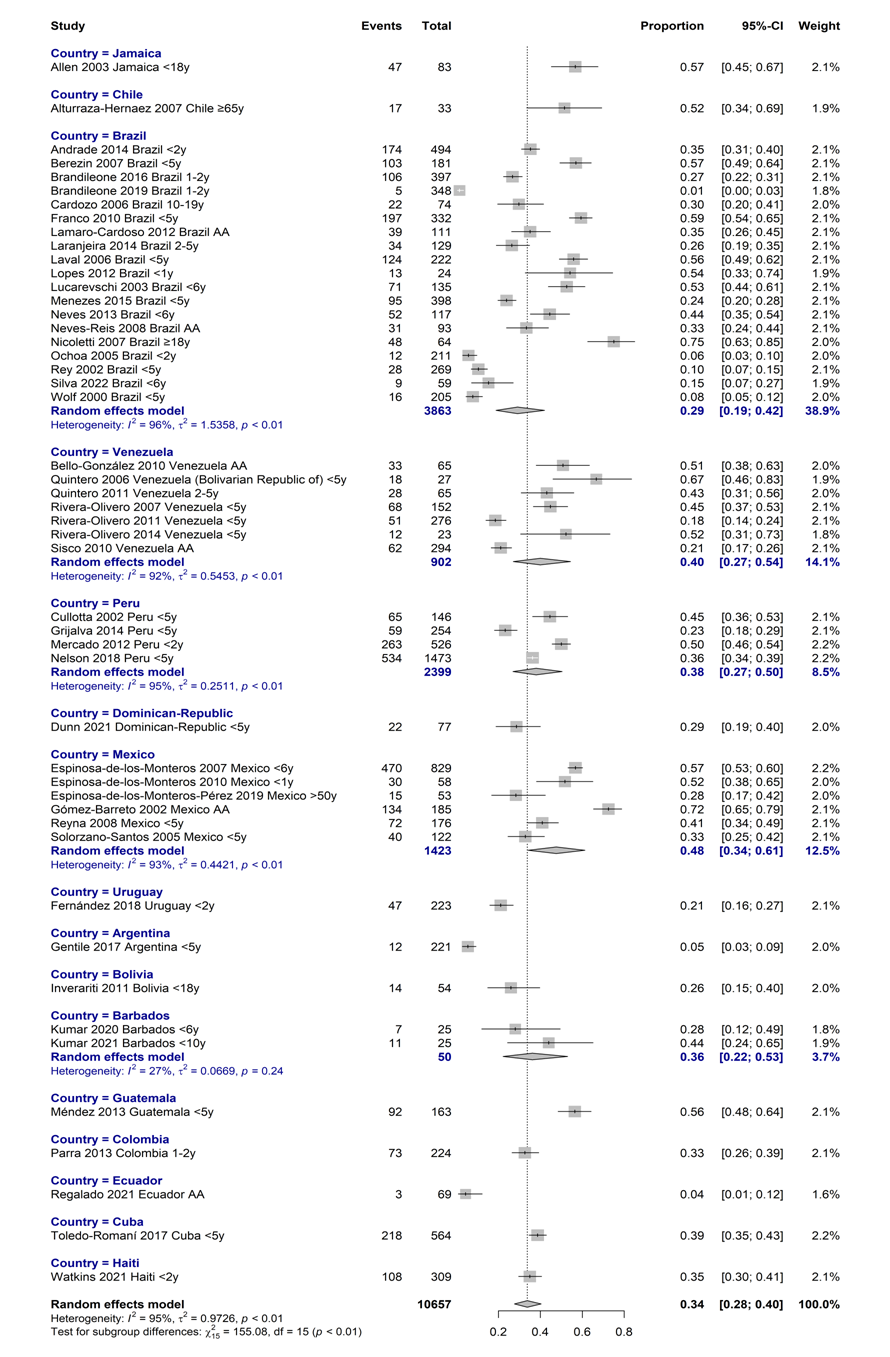


**S5 Figure. Meta-analysis of PCV13 nasopharyngeal carriage**


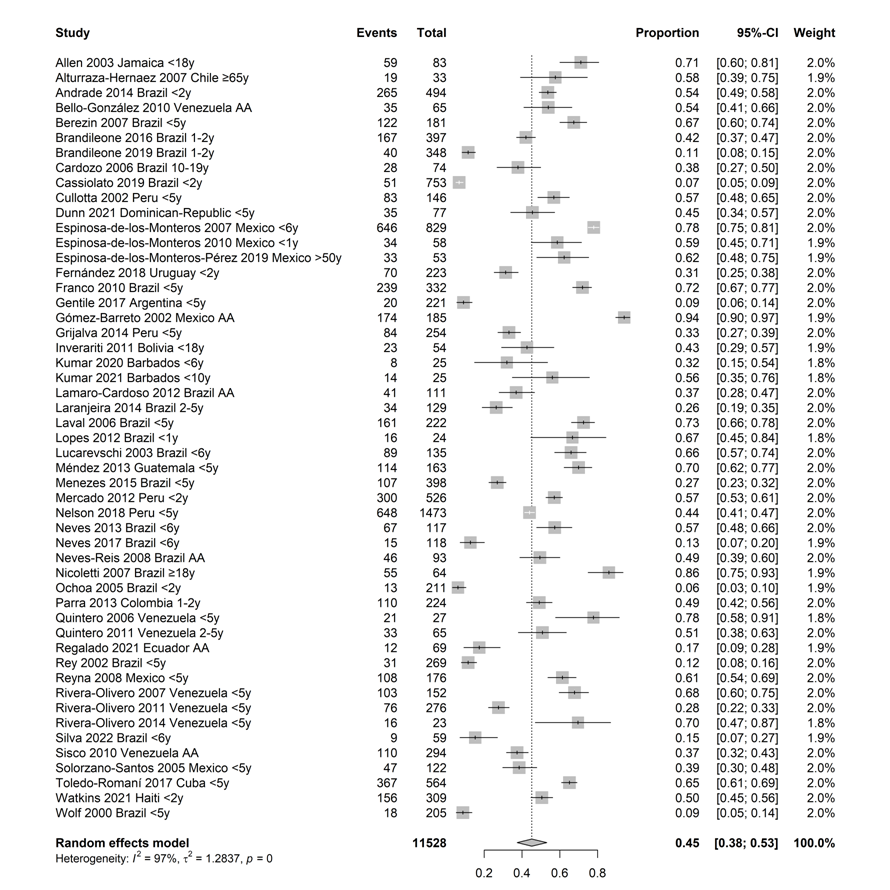


**S6 Figure. Meta-analysis of PCV13 nasopharyngeal carriage by lustrum**


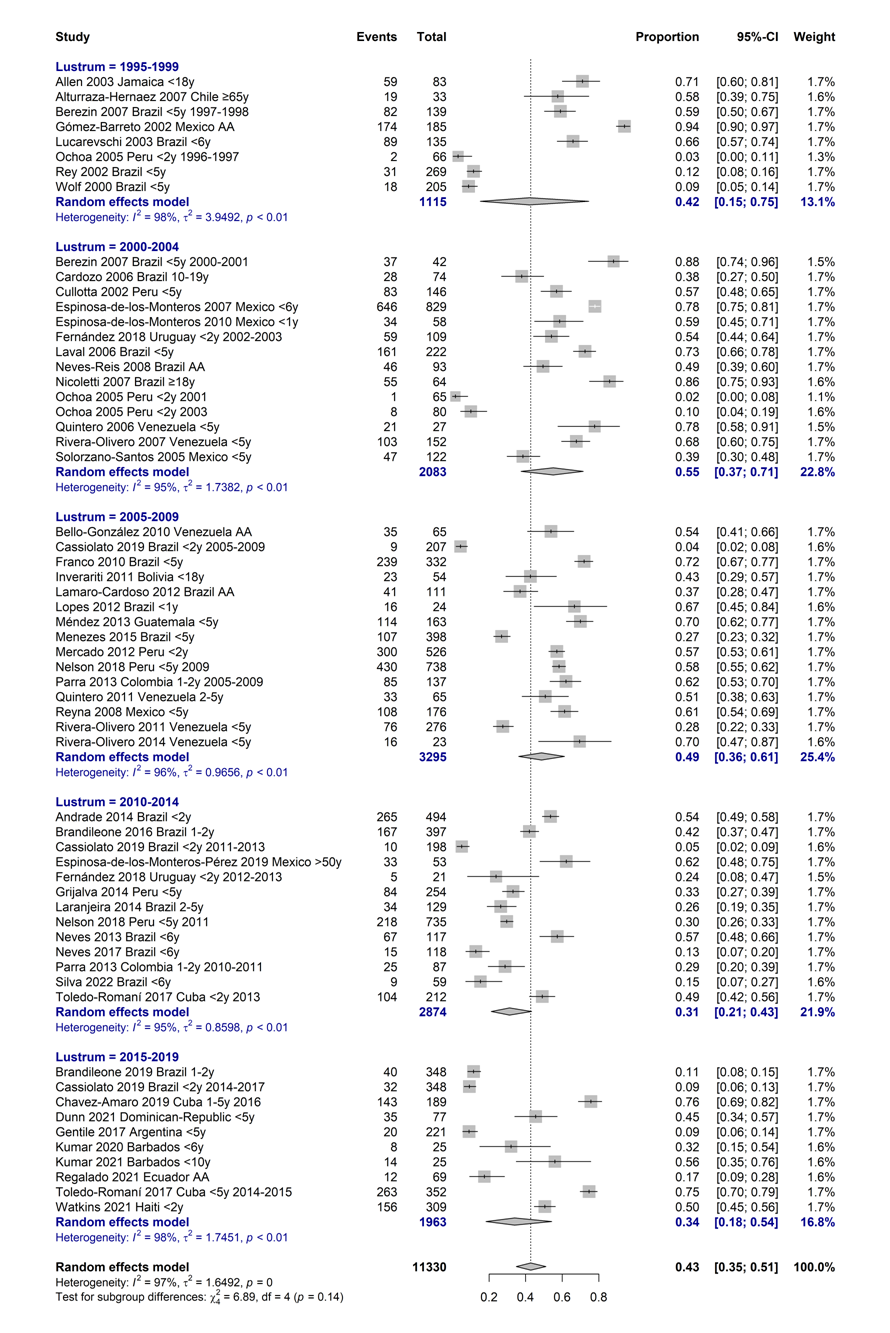


**S7 Figure. Meta-analysis of PCV13 nasopharyngeal carriage by age**
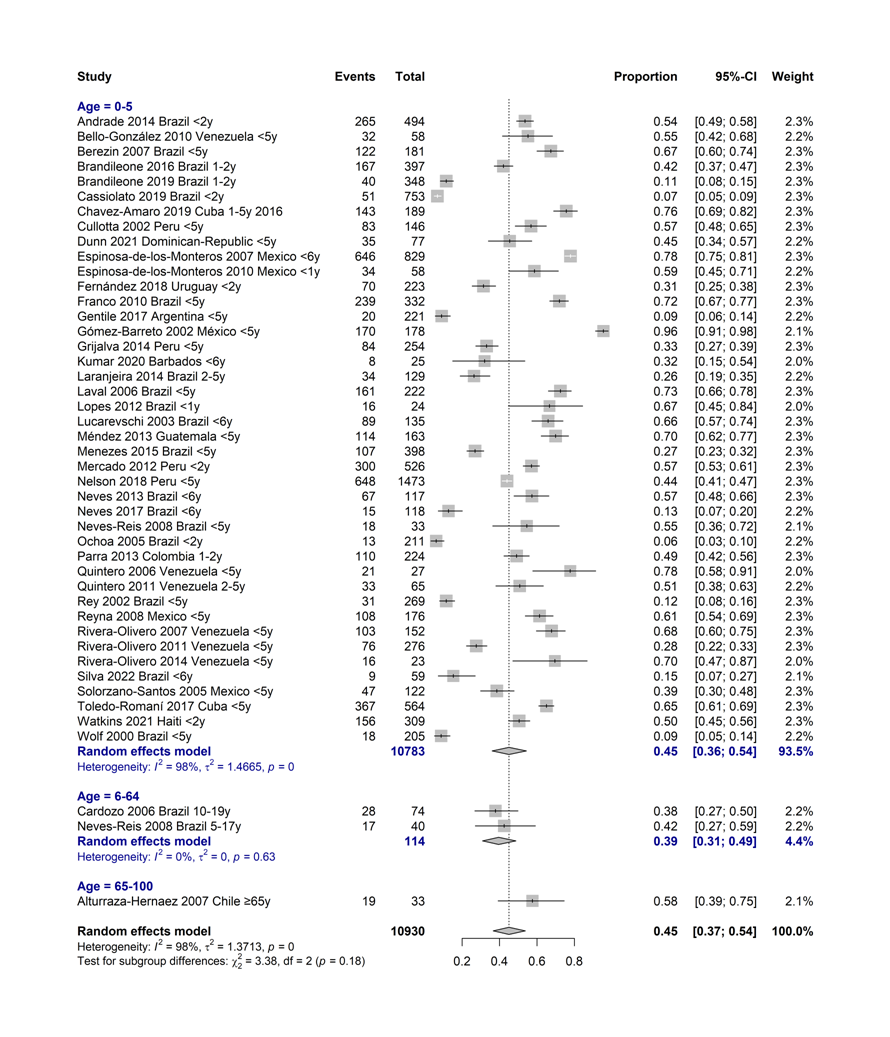


**S8 Figure. Meta-analysis of PCV13 nasopharyngeal carriage by country**


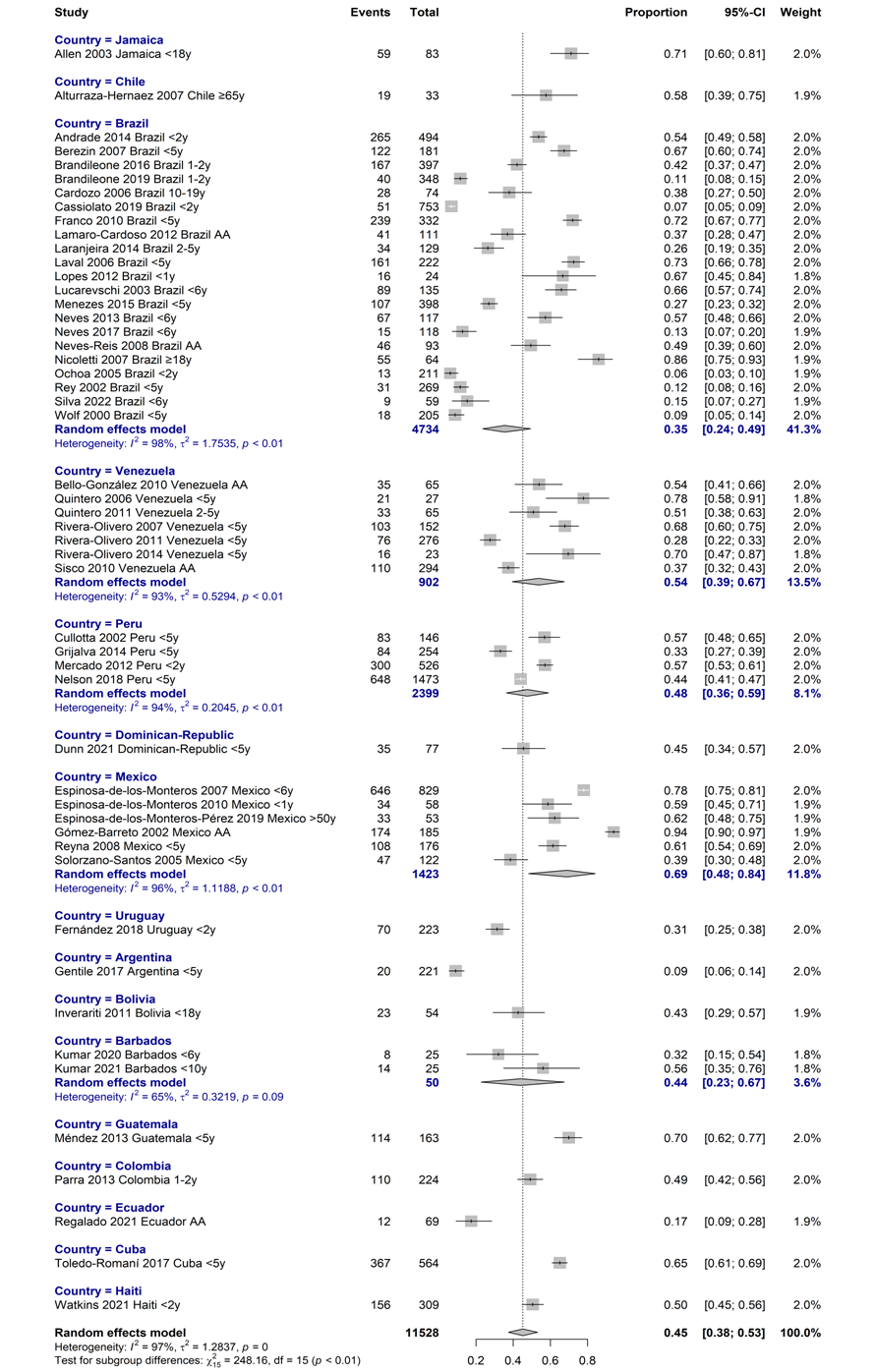

Supplement: S1 File — (DOCX) [file pone.0297767.s001.docx]
