# Supplementary material for: Nasopharyngeal carriage of Streptococcus pneumoniae in Latin America and the Caribbean: A systematic review and meta-analysis
Source: PLoS One. 2024 May 20;19(5):e0297767. doi: 10.1371/journal.pone.0297767 (PMC11104613; doi:10.1371/journal.pone.0297767)
Supplement: Attachment — Submitted filename: Response to Reviewers PONE-D-23-35902.docx [file pone.0297767.s002.docx]

Dear editor and reviewers, thank you very much for your valuable contributions and suggestions. We have followed all suggestions and corrections to improve the quality of our research.

We detail below the response to each of the points and have highlighted the modifications in the manuscript. Again thank you very much.

1. Financial disclosure:

“All authors have received funding by Pfizer Global Medical Grants (GMG) 76436251.”

Please state what role the funders took in the study. If the funders had no role, please state: "The funders had no role in study design, data collection and analysis, decision to publish, or preparation of the manuscript."

If this statement is not correct you must amend it as needed.

Please include this amended Role of Funder statement in your cover letter; we will change the online submission form on your behalf.

Response:

**"The funders had no role in study design, data collection and analysis, decision to publish, or preparation of the manuscript."**

Please include this amended Role of Funder statement in your cover letter. We will change the online submission form on your behalf. **It was made.**

2. Please include a separate caption for each figure in your manuscript.

**A separate caption for each figure was included in the manuscript body.**

3. Table 1: It is best to sort the studies by year of publication, from 2001 to 2022.

**All the included studies were sorted by date.**

4. **All the corrections suggested by Reviewer #1 was made in the manuscript and highlighted.**
